# Tracking leukemic T‐cell transcriptional dynamics *in vivo* with a blood‐based reporter assay

**DOI:** 10.1002/2211-5463.12940

**Published:** 2020-08-12

**Authors:** Alfred G. Tamayo, Syukri Shukor, Alexandra Burr, Patrick Erickson, Biju Parekkadan

**Affiliations:** ^1^ Center for Surgery, Innovation, and Bioengineering Department of Surgery Harvard Medical School Massachusetts General Hospital Shriners Hospitals for Children Boston MA USA; ^2^ Department of Biomedical Engineering Rutgers University Piscataway NJ USA; ^3^ Harvard Stem Cell Institute Cambridge MA USA

**Keywords:** circadian clocks, leukemic T cells, secreted luciferase, transcriptional reporter

## Abstract

Transcriptional dynamics of cancer cells govern cell fate decisions and are therapeutically actionable drug targets. In this study, we engineered a circulating cancer cell line that secretes a luciferase reporter to capture constitutive and circadian clock‐driven transcription dynamics over the course of a day. Engineered human leukemic T cells (Jurkat) were observed to rhythmically secrete luciferase in a continuous flow cell culture system. When transplanted *in vivo,* engineered leukemic cells caused circadian plasma luciferase activity and had expected pathological signs of leukemic disease. This technique is rapid and noninvasive, requiring only a few microliters of media or blood, and can aid in investigating relationships between *in vivo* cancer cell signaling and behavior, such as diet or sleep.

## Graphical abstract

An engineered, circulating cancer cell line secreting a luciferase reporter captures constitutive and circadian clock‐driven transcription dynamics. This was observed *in vitro* in a continuous flow cell culture system, and *in vivo* detecting circadian luciferase plasma activity. This technique is rapid, noninvasive and can aid in investigating relationships between cancer cell signaling and behavior, such as diet or sleep.

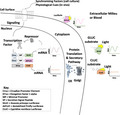

**Abbreviations**
CLUC: *Cypridina noctiluca* luciferase
GLUC: *Gaussia princeps* luciferase

Increasingly, cancer cell interactions with normal physiological environments are known to play an important role in pathology [1]. Solid tumors for example can progress toward malignancy aided by interactions with the tumor‐associated fibroblasts [2, 3], and leukemic cell growth may be enhanced by bone marrow microenvironments [4]. We are only beginning to understand the relationships between oncogenesis and systemic processes, such as sleep [5]. At the cellular level, cancer cells hijack normal signaling pathways to enhance proliferation, angiogenesis, and migration, often by activating or repressing key transcription factors [6]. While technically challenging, real‐time monitoring of signaling pathways in situ provides a valuable window into malignant progression that is difficult if not impossible to capture *in vitro* [7, 8].

In this study, we begin developing a noninvasive assay system to track the transcriptional activity of transplanted leukemic T cells with a resolution of a few hours, using circadian transcriptional regulation as a model. The circadian clock signaling pathway provides predictable transcriptional dynamics within the course of a single day. Circadian clocks are defined by daily rhythms of gene expression in both healthy and cancer cells, and are underexplored in cancer cells within the body [9, 10]. Cancer cell lines are routinely used to study molecules that regulate circadian clocks in cell culture models via transcriptional reporters [11, 12]. Yet by comparison, cancer cell clocks in tumor or xenotransplantation models are rarely studied [13]. Furthermore, clocks are master regulators of cellular metabolism [14] and are linked to the cell cycle [15], and their disruption in certain cell types leads to arrested cancer cell growth [16, 17].

Circadian clocks are composed of complex molecular feedback loops found in tissues across the body [18], driving daily rhythms of transcription and protein expression at the organismal level. A core component of circadian clocks is a transcriptional feedback loop composed of activation and repression protein complexes, known, respectively, as CLOCK‐BMAL1 and the PER complex [19]. CLOCK‐BMAL1 and associated proteins activate the transcription of a large and diverse set of genes, including Per1, Per2, Cry1, and Cry2, which are key components of the PER complex. This results in CLOCK‐BMAL1 repression until the PER complex is degraded; a cycle that repeats approximately every 24 h and can be monitored *in vivo* and in cell culture using native promoter regions of clock‐controlled genes [11, 12, 20]. For this study, we designed synthetic circadian reporters based on our previous biochemical characterization of CLOCK‐BMAL1 DNA binding sequences and other work [21, 22, 23].

To our knowledge, circadian reporter expression has not been observed in acute leukemic T cells to date. Even normal lymphocytes are largely absent from studies characterizing molecular rhythms [24, 25], with few exceptions [26, 27, 28, 29]. This may indicate that leukocytes possess very weak transcriptional rhythms that are difficult to detect. Nonetheless, lymphocyte pathogen response, differentiation, and migration have all been shown to carry circadian rhythms [30, 31, 32].

Here, we show that a human leukemic T‐cell line secretes luciferase rhythmically in cell culture and into the bloodstream of immuno‐compromised mice upon xenotransplantation. We envision that this approach can be used study how signaling pathways interact with cancer therapeutics *in vivo*, requiring only a few microliters of blood. It may also be used to study the interplay between malignancies and systemic disturbances, such as obesity or sleep deprivation.

## Results

### Clock‐driven secretion of luciferase from human leukemic T cells in cell culture

**Fig. 1:**
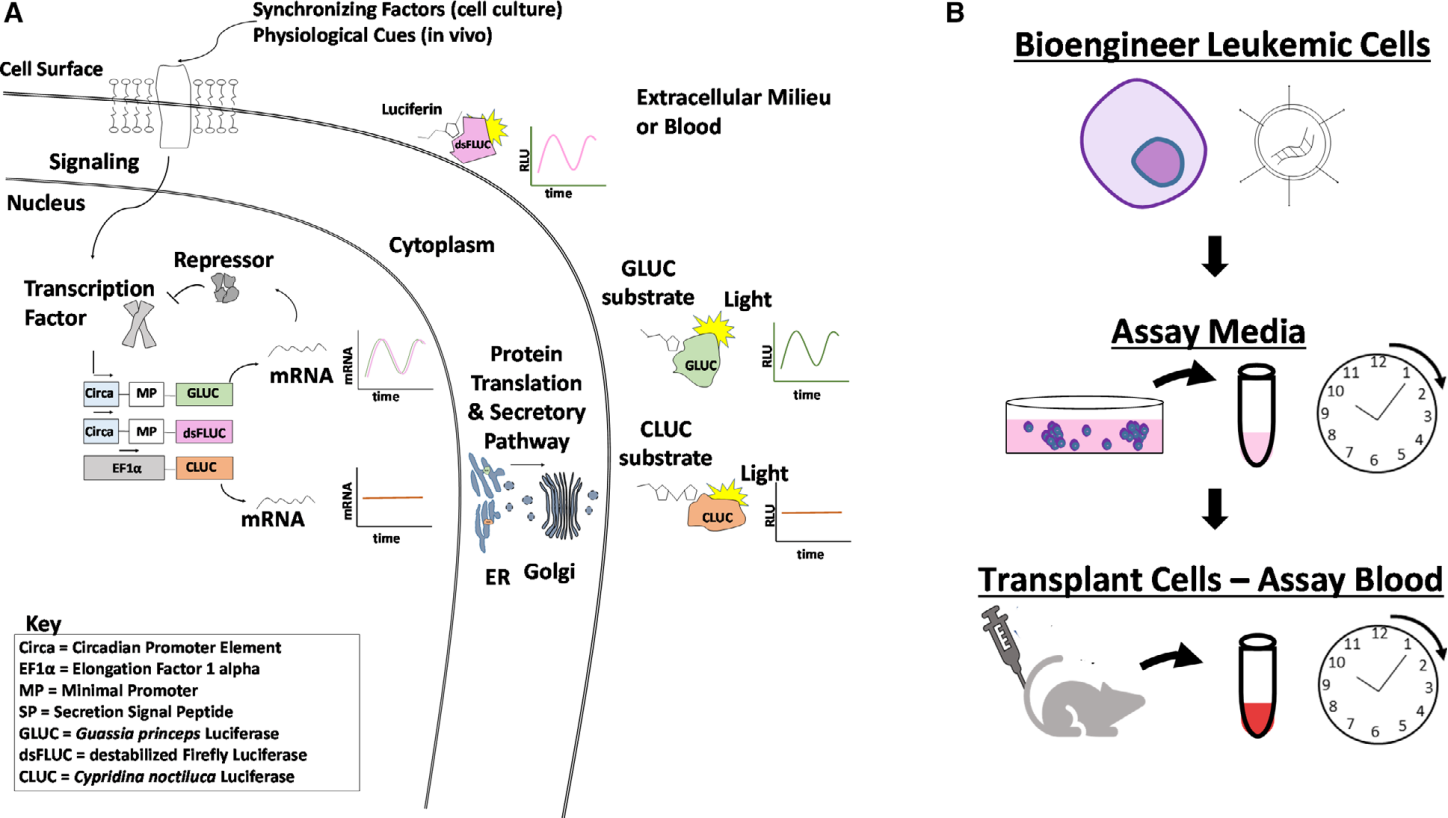
Illustration of luciferase reporters and experimental approach used in this study. (A) Illustration of constitutive and circadian secretion of secreted luciferases CLUC and GLUC, versus cytoplasmic destabilized firefly luciferase. (B) Schematic of experimental approach. Recombinant DNA was introduced into lymphoblastic leukemic T cells (Jurkat). Secretion of luciferases in the media *in vitro* was monitored for 24–48 h. Cells were engrafted into animals, and blood was assayed for luciferase over the course of a day.

Destabilized firefly luciferase, which is largely cytosolic, has traditionally been used to monitor circadian clocks [11, 12]. Yamaguchi et al. first showed *in vivo* recording of central clocks in mice using this firefly luciferase system [33]. *Gaussia princeps* and *Cypridina noctiluca* luciferases, GLUC and CLUC, respectively, [34, 35] are naturally secreted forms of luciferase and allow for easy, real‐time monitoring *in vivo* through blood sample collection (Fig. 1A). GLUC has been used in previous studies to monitor cell expansion of solid tumor xenografts in living animals and has a half‐life of ~ 20 min in mouse plasma, allowing for the monitoring of dynamic secretion [7, 36]. In fact, these luciferases have been used to track circadian expression in the plasma of transgenic mice and in fibroblasts *in vitro* [37, 38]. GLUC and CLUC have been shown to catalyze distinct substrates to generate light, which allows for their use as dual luciferase reporters [39]. We engineered human leukemic T cells (Jurkat E6‐1) to express GLUC and CLUC from constitutive and circadian clock response elements (RE) (further discussed below) to first detect secretion rhythms in cell culture before testing them in a xenotransplantation model (Fig. 1B).

We used EF1α, a strong, constitutive promoter to validate our assays as a reference promoter, and as an indicator of basal transcription and protein synthesis [40]. Jurkat human leukemic T cells were transduced and made stable by negative (blasticidin) and/or positive (GFP) selection. Stable cell lines or parental Jurkat cells (untransduced) were then incubated in standard media for 2 h, at which point media was collected and assayed using either GLUC substrate (coelenterazine) or CLUC substrate (Fig. 3A,B). While positive signals were several orders of magnitude above background, we observed no significant difference in signal between untransduced and CLUC expressing cell‐conditioned media when incubated with GLUC substrate, and the converse was true for GLUC‐expressing cell‐conditioned media incubated with CLUC substrate. These results confirm that the Jurkat cell secreted GLUC and CLUC maintain exclusive substrate specificity.

Luciferase enzymes have long been a staple choice for monitoring gene transcriptional reporters due to their extraordinary sensitivity and dynamic range [41]. We characterized the ability of GLUC secretion to correlate with cell number by conditioning media with varying concentrations of stably secreting Jurkat cells (EF1α‐GLUC). Remarkably, cell concentrations of < 1000 cells per mL (Fig. S1A) and subpicogram amounts of GLUC could be detected with linear correlations (Fig. S1B). We developed lentiviral constructs with constitutive and circadian promoter elements driving GLUC or CLUC expression and showed that these constructs were competent for transduction using GFP markers (Fig. S3A). Additionally, GLUC expression did not affect cell growth parameters (Fig. S3B). The ultra‐sensitivity of GLUC should allow for the detection of a very small number of actively secreting cells, a potential constraint in animal models.

To characterize the dynamic nature of RE secretion over at least 24 h, we created a system to flow media over sediment suspension cells followed by automated collection of media (Figs 3C and S6). To examine constitutive reporters, we transduced Jurkat cells with constructs driving GLUC and CLUC expression from CMV and EF1α promoters, respectively. Cells were seeded onto an air permeable tubular vessel and allowed to settle for 3–4 h before flowing media through the vessel at 3.8 mL·h^−1^, and media outflow was collected at a rate of one fraction per hour. Individual fractions were then assayed (20 μL of 3.8 mL fractions) for CLUC and GLUC activity. Preliminary experiments demonstrated that a few hours are needed for media to circulate through the flow collection system and stabilize before showing secreted protein levels (data not shown). To prevent this artifact of the media collection system in data analysis, we discarded the first few hours of media collection. In order to compare rhythmicity on similar scales of amplitude, each raw data set was then normalized to the mean of all data points and set to 0 and linear detrended before plotting both GLUC and CLUC data sets on a single graph (Fig. 3D). These results demonstrate that both CMV and EF1α promoters drive stable expression with minimal fluctuation over a 24‐h period.

The core circadian clock RE consists of a palindromic 6‐base pair sequence known as the E‐box [18]. E‐box sequences are high‐affinity binding sites of the CLOCK‐BMAL1 transcription factor heterodimer within clock‐regulated gene promoters. Studies have shown that E‐box sequences from promoter regions of clock‐regulated genes are sufficient to drive rhythmic gene expression [11, 12]. RE compactness was a factor we considered since large RE‐gene cassettes may hinder viral production, and poorly characterized DNA sequences may be a source of noise or toxicity. We based the designs of clock REs on our previous work showing that three to six much abbreviated portions of the Per1 gene promoter containing E‐box sequences (16–21 base pairs) specifically bind CLOCK‐BMAL1 and recapitulate circadian promoter binding *in vitro* [22, 23]. This synthetic clock reporter, we call Circa2, contains six E‐box sequences, respectively, flanked by 12 base pairs of Per1 promoter sequence and followed by a short 31 base pair minimal promoter (MP) base pairs, with total size of 144 base pairs.

**Fig. 2:**
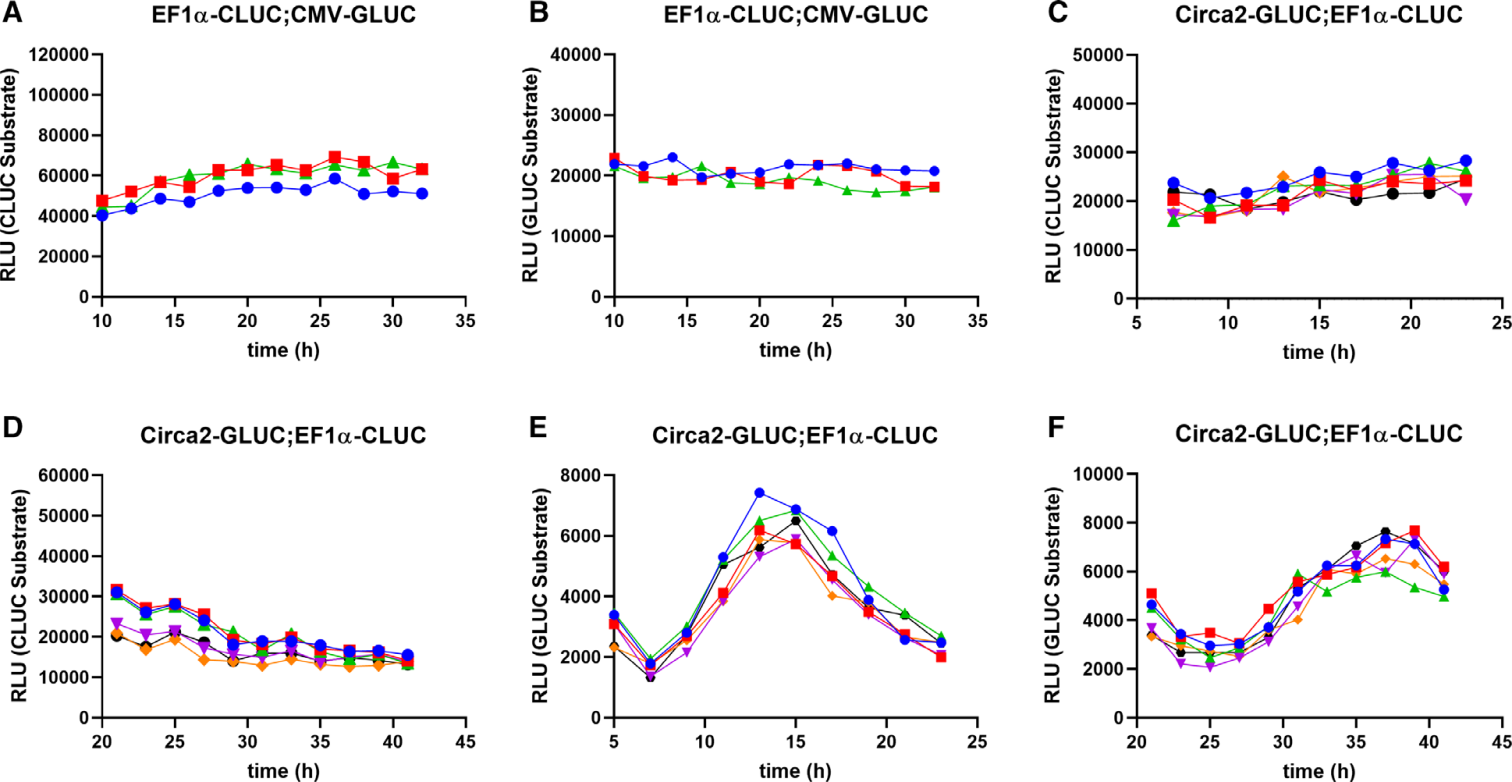
Underlying data for rhythmic and constitutive secretion of GLUC and CLUC from Jurkat cells in a suspension cell flow collection system. Jurkat cell secretions were collected from synchronized EF1α‐CLUC;CMV‐GLUC and assayed for (A) CLUC or (B) GLUC. Circa2‐GLUC;EF1α‐CLUC cell secretions were collected beginning 7 h postsynchronization and assayed for (C) CLUC or (E) GLUC, or beginning 23 h postsynchronization and assayed for (D) CLUC or (F) GLUC. Each line represents a replicate (*N* = 6).

**Fig. 3:**
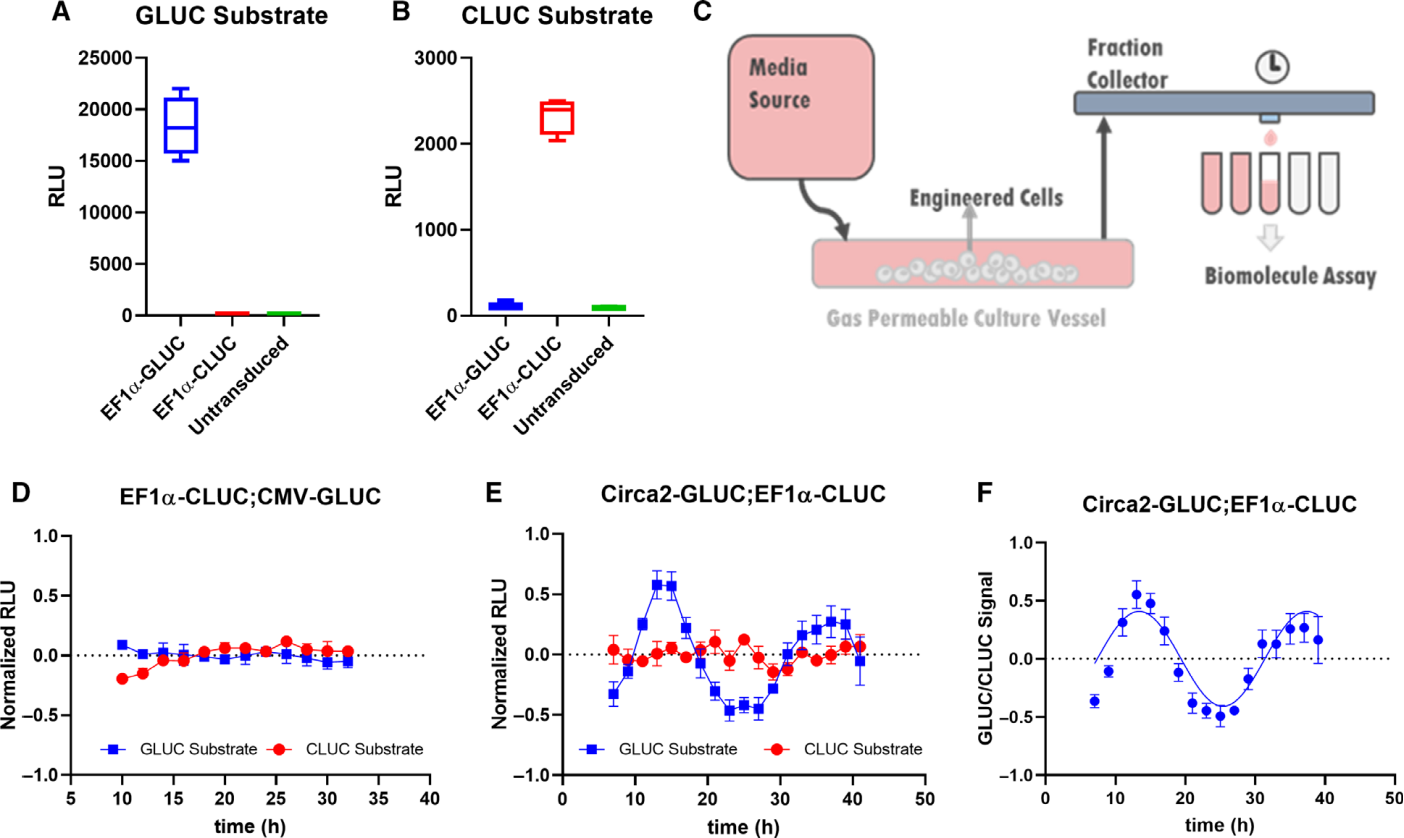
Rhythmic and constitutive secretion of GLUC and CLUC from lymphoblastic leukemic T cells in a suspension cell flow collection system. Media conditioned with EF1α‐GLUC, EF1α‐CLUC, or unmodified (untransduced) Jurkat cells was assayed using (A) GLUC substrate or (B) CLUC substrate separately. (C) Schematic of a continuous flow system in which Jurkat cell secretion was monitored. Jurkat cell secretions were collected from synchronized (D) EF1α‐CLUC; CMV‐GLUC or (E) Circa2‐GLUC; EF1α‐CLUC cells, linear detrended (Period: ~ 21.3 h), and amplitude normalized (SD; *N* = 6). (F) Circa2‐GLUC signals were normalized to EF1α‐CLUC signals (quotient of amplitude normalized and linear detrended GLUC to CLUC signals) and an undamped 24‐h Sine wave was fitted to the data (Prism, least squares method, degrees of freedom: 14, R squared: 0.7777, sum of squares: 0.4124). Student's *t*‐test was used to assess significance where relevant.

Lentiviral plasmids containing Circa2 driving GLUC expression were electroporated into Jurkat cells constitutively expressing CLUC from the EF1α promoter. Cells were allowed to recover overnight before synchronizing with dexamethasone treatment for 30 min [42], washing then loading onto the laminar flow system as described above. Fractions were collected every hour and analyzed for GLUC and CLUC activity with a 2‐h resolution, and raw data were normalized to the mean of all data points, detrended, and set to 0 as before (Fig. 3E). Expression from constitutive promoters resulted in nonrhythmic fluctuation of GLUC and CLUC expression/secretion, at least some of which we assume is experimental noise inherent to our flow collection system (Fig. 2C), with seemingly little effect on rhythmic GLUC secretion. To illustrate this point, Circa‐2‐driven GLUC data were normalized to EF1α driven CLUC to which sine curve with a 24‐h wavelength constraint could be fitted with confidence (Fig. 3F). These normalized data were used to calculate an approximate period length of 21.3 h. These results demonstrate that circadian promoter elements can drive rhythmic gene expression and secretion from human leukemic T cells. Raw data plots of CLUC (Fig. 2A,C,D) and GLUC (Fig. 2B,E,F) are shown for cells expressing GLUC from Circa2 and CLUC from EF1α or CMV, and expectedly revealed higher concentrations of GLUC secreted from EF1a promoters than from Circa2/ MP constructs. Dynamic expression of GLUC from Circa2 was also verified in a U2OS cell line, which is known to have strong rhythms (Fig. S2). The results from U2OS studies suggest that our Circa2 construct, although active, can be further codon‐optimized to have more robust rhythmicity in the future.

**Fig. 4:**
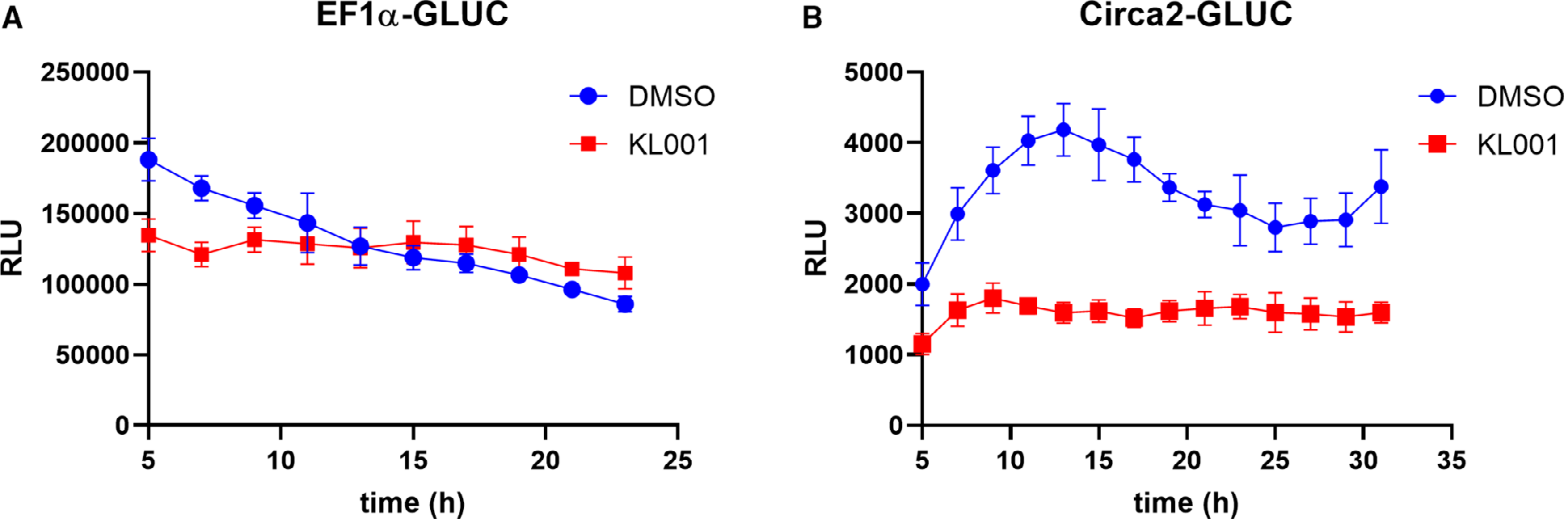
Pharmacological disruption of circadian clocks abolishes dynamic nature of Circa2 driven GLUC secretion in leukemic T cells. (A) EF1α‐GLUC or (B) Circa2‐GLUC cells were treated with DMSO (vehicle) or 20 μm KL001 for 24 h prior to synchronization and seeding for cell secretion analysis (GLUC substrate) using continuous flow (SD; *N* = 3). Student's *t*‐test was used to assess significance where relevant.

To confirm that the oscillatory nature of gene expression was circadian clock related, we next treated cells with a pharmacological agent previously shown to disrupt circadian clocks. KL001 has been shown to stabilize CRY1 protein, a CLOCK‐BMAL1 repressor, leading to a dysfunctional transcriptional feedback loop [43]. Stable Jurkat cell lines expressing GLUC driven by Circa2 or EF1α as a reference were treated with a sublethal dose of KL001 (20 μm) or vehicle (DMSO) for 24 h before dexamethasone synchronization and seeding on the laminar flow system as described above. KL001 treatment had little to no effect on EF1α‐driven GLUC secretion as shown by raw and normalized data (Fig. 4A), while severely disrupting the dynamic nature of Circa2‐driven GLUC secretion (Fig. 4B). These results show that the secreted reporter system is sensitive to drugs targeting the circadian clock and can be useful for further evaluation of targeted agents.

To better understand the nature of Jurkat cell endogenous clocks, we measured the expression of core clock‐controlled genes every 2 h upon synchronization with dexamethasone in untransduced Jurkat cells. We show by RT‐qPCR that Per1, Per2, Bmal1, Clock, Cry1, and Cry2 mRNA transcripts were all detectable, suggesting a rhythmic pattern of expression for Per1, Per2, Bmal1, and Clock (Fig. SA‐F). Per1 and Per2 peaked at around 12 h postsynchronization (Fig. S4G), consistent with our reporters which are based on promoter elements found in those genes. Clock and Bmal1 are controlled by a different arm of the clock [18] and expectedly yielded an anti‐phasic pattern of expression, peaking nearly 12 h earlier than Per1 and Per2. The differences in mRNA expression between the peaks and troughs of Per1, Per2, and Bmal1 were statistically significant (Fig. S4H). It is unclear why Cry1 and Cry2 expressions rhythms were too weak to observe. There results demonstrate that endogenous circadian genes are weakly rhythmic at the mRNA level in Jurkat cells, consistent with our observations using secreted luciferase reporters.

### Constitutive and dynamic luciferase secretion from xenotransplanted cells in mice

**Fig. 5:**
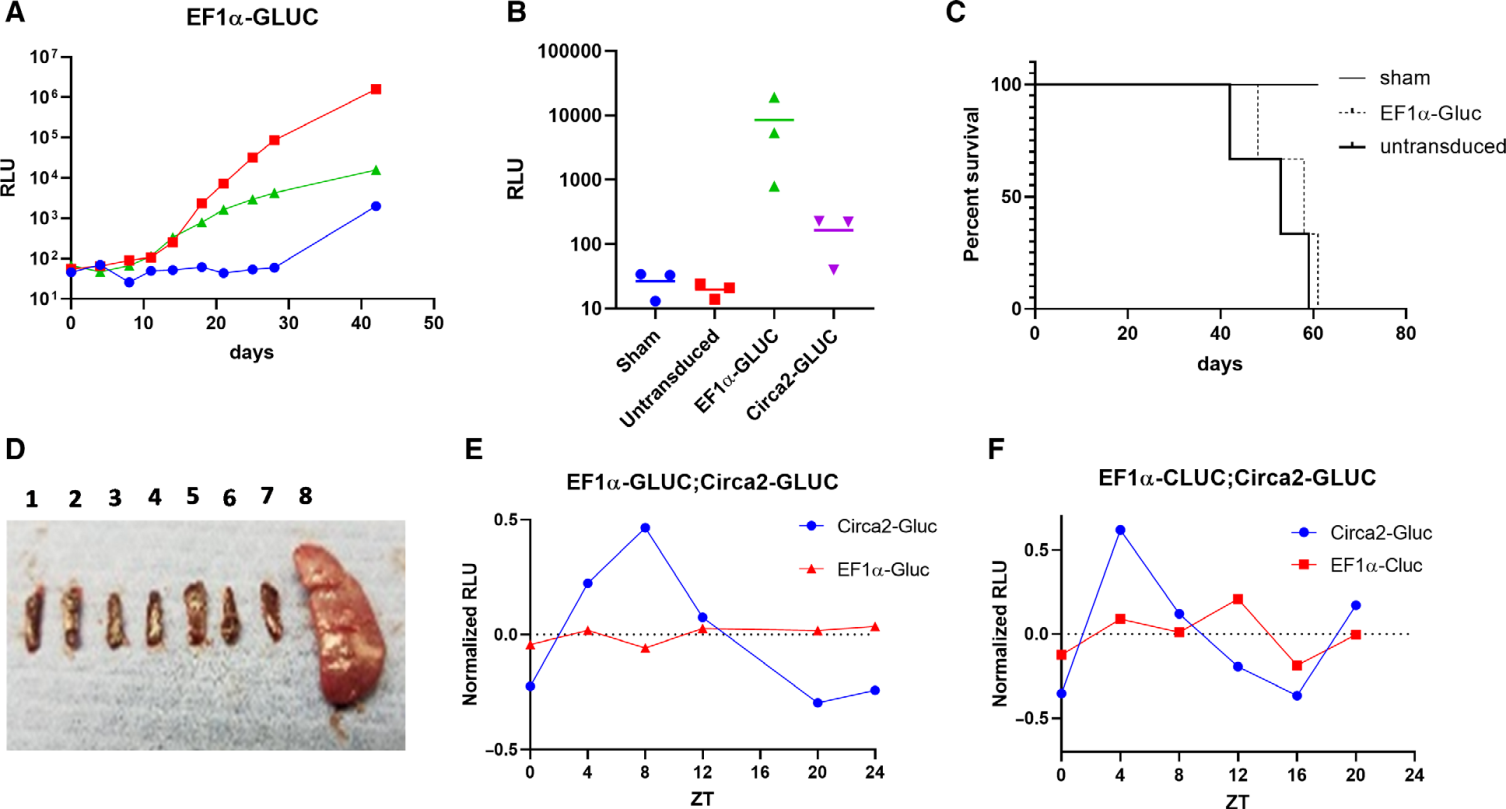
Constitutive and circadian clock reporter dynamics of leukemic T cells infiltrating immuno‐compromised mice. (A) Blood was collected from NSG/SCID mice were injected with EF1α‐GLUC Jurkat cells every few days and assayed for GLUC (5 μL blood). Each marker represents an individual mouse. Time 0 was measured before mice were injected. (B) Mice were injected with sham (PBS vehicle), unmodified (untransduced), EF1α‐GLUC, or Circa2‐GLUC Jurkat cells, and blood was assayed or GLUC 40 days later. Each marker represents a single mouse. (C) Survival curve of mice injected with sham (PBS vehicle), EF1α‐GLUC, or unmodified (untransduced) Jurkat cells over a period of 61 days. (D) Spleens were collected from mice injected with sham (1,2), EF1α‐GLUC (3,4,5), and untransduced (6,7,8) Jurkat cells. (E) Blood was collected approximately every 4 h from mice injected with either EF1α‐GLUC or Circa2‐GLUC Jurkat cells and assayed for GLUC. (*N* = 2). (F) EF1α‐CLUC and Circa2‐GLUC Jurkat cells were co‐injected into a single mouse, and then, blood was collected every 4 h and assayed for GLUC and CLUC. Lights on at ZT0 and lights off at ZT12. Raw RLU counts (5 μL blood) were amplitude normalized. Student's *t*‐test was used to assess significance where relevant.

We next set out to determine whether clock‐driven secretion from human leukemic T cells would persist in animals upon transplantation. We first used EF1α driven GLUC expression to determine the optimal time post‐transplantation for data collection. Stably transduced Jurkat cells were injected into immune‐compromised mice (NSG), and plasma GLUC activity was monitored (5 μL plasma assayed) for 42 days post‐transplantation. Animals expressed plasma EF1α‐driven GLUC levels above prebleed levels (time 0) between 10 and 30 days with variable kinetic profiles (Fig. 5A). Animals injected with Circa2‐GLUC expressing cells required at least 40 days to express plasma levels of GLUC above background (Fig. 5B), likely because they are expressing far lower levels of GLUC, as predicted by our *in vitro* observations. We estimate that these signals correspond to between ~ 200–0.1 ng·mL^−1^ of GLUC per mL of blood based on a GLUC standard curve.

The delay in plasma GLUC concentration when driven by Circa2 presented a challenge because mice transplanted with Jurkat cells began showing signs of disease around 43 days post‐transplantation, at which point they were euthanized in accordance with the animal protocol. About 30% of animals transplanted with Circa2‐GLUC cells were healthy enough to undergo further experiments when plasma GLUC concentrations were detected. Most animals were euthanized by 61 days, with one animal found dead at 43 days (Fig. 5C). We observed variable cancer associate pathologies including lower limb paralysis, lethargy, weight loss, tumors, and a case of splenomegaly (Fig. 5D).

Mice (NSG) were injected with stably selected Jurkat cells expressing GLUC from the Circa2 synthetic clock RE or from the EF1α promoter as a reference. Plasma GLUC signals were measured every 4 h for 24 h. We observed that Circa2‐driven GLUC levels in plasma have a dynamic profile consistent with circadian clock‐dependent transcription, as compared to EF1α‐driven secretion (Fig. 5E). Raw data points were normalized to the mean signal set to 0, averaged for each set. In a separate experiment, immuno‐compromised mice (NSG) were injected with two different stably selected cell lines expressing GLUC from Circa2 or CLUC from EF1α as an internal reference. Similarly, EF1α‐driven plasma CLUC signals were stable as compared to Circa2‐driven GLUC signals (Fig. 5F). These data clearly demonstrate the potential use of this approach to study transcriptional dynamics of cancer cells *in vivo*, though optimization efforts would be required to improve the consistency of results.

Furthermore, given that our cell culture results indicate that the clocks of Jurkat cells are not strongly coupled, these experiments suggest that Jurkat human leukemic T cells synchronize to mouse physiological time‐setting cues. As shown in Fig. 5E,F, the secretion of clock‐driven cells peak around hour 4–8 ZT and are at a minimum around hour 16–20 ZT. Underlying data for circadian time‐dependent analysis of plasma GLUC and CLUC signals are also reported (Fig. S5).

## Discussion

Interactions between normal physiological systems and cancer cell signaling pathways play an important role in cancer pathology [1, 6], which is difficult to capture in cell culture models. In this study, we engineered human leukemic T cells (Jurkat) to secrete luciferase reporters in order to track their transcriptional behavior in a mouse transplant model over the course of a day (Fig. 3A,B). We first set out to test this approach *in vitro* using constitutive and circadian clock reporters, expecting a clear juxtaposition between these reporters. Indeed, when driven by circadian promoter elements (Circa2) instead of constitutive promoters, we observed media luciferase rhythms consistent with ~ 24‐h cycles of clock‐driven gene expression (Fig. 3E,F). Our data suggest that Jurkat leukemic T cells may have weak and poorly coupled circadian clocks, corroborating with the weak oscillatory nature of endogenous clock gene expression (Fig. S4H), consistent with previous observations [26]. These are clock‐driven rhythms could also be pharmacologically disrupted using the small molecule KL001 (Fig. 4B).

We then transplanted engineered Jurkat leukemic T cells into immuno‐compromised mice and monitored blood luciferase levels using only a few microliters of plasma. Leukemic T cells are an attractive model to test this approach, since they are likely less amenable than solid tumors to traditional luciferase reporter assays using imaging techniques. We expected that the blood cancer cells might infiltrate a variety of tissues upon transplantation, especially bone marrow and spleen [44, 45]. The nature of the pathologies we encountered post‐transplantation were varied, with for example one out of six mice developing a severely enlarged spleen (Fig. 5D). The oscillatory nature of luciferase secretion was consistent a circadian pattern of rhythmicity (Fig. 5E,F). These studies were not performed under constant darkness, and thus, it is not clear if circadian rhythms are driven by circadian clock of animals or light–dark time cue. Though, given that Jurkat clocks likely decouple soon after transplantation, this raises the interesting possibility that human leukemic T‐cell clocks are synchronized by normal mouse time‐setting cues. And that these cues are independent of a fully functional immune system. More broadly, the general mechanisms by which peripheral clocks are synchronized to the central brain clock remain unclear; a question this experimental system may be used to explore.

We envision that future applications of this approach will open a window into cancer cell signaling events technically difficult to monitor. Reporter cells could be deployed in the context of systemic changes that cannot be recapitulated *in vitro*, such as diet and sleep disturbances. Besides clocks, well‐characterized transcriptional reporters may be implemented, such as those for Nf‐κB signaling [7], which has shown potential as a therapeutic target for leukemia [46]. Secreted reporters may be used to study acute cancer cell responses to drug treatments, for example, perhaps offering clues to drug resistance mechanisms that would otherwise go unnoticed. And while this study describes engineered cells prior to transplantation, it may be possible to engineer cancer targeting viruses with secreted reporters, opening more experimental avenues.

## Materials and methods

### Plasmid construction

Circa promoters are synthetic circadian REs based on our previous work [22, 23], upstream of a MP. Source DNA sequence for MP was pGL4.24[luc2P/minP] (Promega, Madison, WI). Sequences can be found in Table S1. Source DNA sequences for constitutive promoter/enhancers were pENTR‐5‐EF1ap (Thermo Fisher Scientific, cat # A11145, Waltham, MA) and pMAXGFP (Lonza, Basel, Switzerland) for EF1α and CMV, respectively. Source DNA sequences for reporters were pCMV‐Gluc2 (New England Biolabs cat # 8081S, Ipswich, MA) and pCMV‐Cluc2 (New England Biolabs cat # N0321) for GLUC and CLUC, respectively. PCR amplicons or custom‐synthesized double‐stranded DNA fragments (Integratied DNA Technologies, Coralville, IA) or promoters and reporters were cloned into pENTR TOPO‐TA (Thermo cat # EP0402) and pENTR d‐TOPO (Thermo cat # K252520) entry vectors, respectively. Multisite Gateway cloning into a promoterless lentiviral plasmid pLenti6.4⁄R4R2⁄V5‐DEST (Thermo cat # A11146) with desired promoter/reporter combination using LR Clonase enzyme mix II (Thermo cat # 11791100) according to manufacturer's instructions. DNA was isolated using Pureyield (Promega cat # A1222) and Purelink (Thermo cat # K210017). Standard procedures were performed to verify clones, including PCR, restriction enzyme digestion and sequencing. Clones were further selected on their ability to express reporters when transfected using Lipofectamine 3000 (Thermo cat # L3000015) in 293t cells (ATCC cat # CRL‐3216, Manassa, VA).

### Cell engineering

Jurkat E6‐1 cells (ATCC cat # TIB‐152) are derived from T lymphocytes originally isolated from a child patient with acute T‐cell leukemia [47]. Jurkat cells were passaged as indicated by vendor with RPMI medium. Lentiviral particles were produced using a protocol modified from the manufacturer of Lipofectamine 3000. Briefly, 293t cells grown to 85–95% confluency in a 75–cm^2^ flask (Corning, NY) were transfected with 2 μg pVSVG, 5 μg pPMDL, 2 μg pRSV and 10 μg lentiviral transfer plasmid using Lipofectamine 3000 in OptiMEM (Thermo cat # 51985091) containing 5% FBS. Lentiviral particles were harvested 24–48 h post‐transfection, spun at 5000 ***g***, filtered through a 40‐μm basket filter (Millipore, Sigma, Burlington, MA), concentrated at 112 398 ***g*** using a Beckman swing bucket rotor (SW‐28) and resuspended in OptiMEM without FBS. Functional titers were determined by transducing 293t cells. Transduced Jurkat cells were selected by blasticidin (Thermo cat # R21001) resistance or by fluorescence‐activated cell sorting (FACS). For blasticidin selection, cells were incubated in media containing 10 μg·mL^−1^ blasticidin for 5 days, and media was replaced with fresh blasticidin containing media as needed for 2 weeks. CMV‐GLUC;EF1α‐CLUC cells were generated by transducing EF1α‐CLUC blasticidin‐resistant cells with Gluc‐IRES‐eGFP (Partners Research Viral Vector Core: Boston, MA, USA) and selecting for GFP positive cells by FACS (sorting was performed by the HSCI‐CRM Partners Research Core Facility: Boston, MA). Circa1/2‐GLUC;EF1α‐CLUC cells were generated by electroporating EF1α‐CLUC blasticidin resistant cells with Circa1/2‐GLUC lentiviral transfer plasmids using an ECM 399 electroporator (BTX). In a 2‐mm cuvette (BTX), 3 × 10^6^ cells resuspended in 200 μL OptiMEM were electroporated with 8 μg lentiviral transfer plasmid DNA at 500 V, 700 µs, single pulse, then immediately resuspended in standard media. Cells recovered for 24 h prior to further experiments.

### Monitoring secretion of luciferase

For both GLUC and CLUC assays, up to 20 μL of conditioned media from engineered cells, mouse plasma, or purified GLUC (Nanolight Technologies cat # 321, Pinetop, AZ) was loaded onto a 96‐well black plate prior to adding working concentration of substrate. A volume of 100 μL of GLUC substrate (coelenterazine) (Nanolight cat # 303‐500) or CLUC substrate (Cypridina Luciferase Substrate) (Nanolight cat # 305‐500) at a concentration of 12.5 ng·mL^−1^ in PBS were added to wells and immediately read with microplate reader (Biotek Synergy 2) with an integration time of 0.1 s. Luminescence was recorded by the device as relative luminescence units (RLU). Continuous monitoring of plasma luciferase from mice is described in a separate section below. To monitor continuous secretion of luciferase from cells *in vitro* over time, cells were seeded onto a custom‐made constant flow cell culture and collection device (Fig. S6). For each experiment, 16 × 10^6^ cells in ~ 2 mL of media were injected into a tube‐like bag made from air permeable material (Rogers Corporation HT‐6240 Transparent 0.010”, Chandler, AZ) with approximate dimensions of 20 cm in length and 0.6 cm in diameter. We have previously shown that vessels made from this material are optimal for the expansion of human T cells [48]. Silicone tubing (Platinum LS14 Masterflex cat # 96410‐14) was used to connect the cell containing vessel to media filled syringes operated by a PHD 2000 syringe pump (Harvard Apparatus, Holliston, MA) and to a Biologic Biofrac fraction collector (Bio‐Rad Technologies, Hercules, CA). Cells were allowed to settle for 3–4 h prior to flowing media at 3.8 mL·h^−1^. Fractions were collected every hour and 20 μL of every other fraction was assayed for GLUC and/or CLUC using a maximum of 12 fractions per 24‐h period of collection. For circadian experiments, cells were synchronized with 10 μm dexamethasone (MilliporeSigma cat # D4902) for 30 min, washed three times with PBS and seeded onto a continuous flow vessel either immediately or after a 24‐h incubation. For KL001 (MilliporeSigma cat # SML1032) experiments, 1 × 10^6^ cells per mL EF1α‐GLUC or Circa2‐GLUC stable cells selected by blasticidin resistance were incubated with 20 μm KL001 or DMSO (vehicle) for 24 h prior to washing, synchronization and continuous flow monitoring as described above. Underlying RLU data was normalized to 0 by dividing raw data points by the average of the data set, then subtracting quotient by the average of the data set. All line fitting was performed using prism 8 (GraphPad, San Diego, CA). Linear detrending and period determination of circadian data was performed using BioDare2 [49].

U2OS cells (ATCC cat # HTB‐96) cells were passaged as indicated by the vendor in McCoy's 5a Medium Modified with 10% FBS (Gibco, Gaithersburg, MD) and 1% Antibiotic Antimycotic Solution (Corning). Passage 3 cells were thawed from nitrogen and plated in 10 mL of medium in a Falcon 75 cm^2^ flask (Corning). The next day, the cells were transfected with the circadian reporter plasmid by electroporation using the Neon Transfection System. Cells were suspended at 10^7^ cells per mL in Resuspension Buffer R (Neon) with plasmid DNA (Plenti GFP SCP2 Gluc) at 1 µg·µL^−1^, and electroporated with four 10 ms pulses at 1230 volts in a 100 µL Neon Pipette tip. Cells were immediately plated in 1 mL of medium at 1.68 × 10^5^ cells per well in a Costar 12‐well plate (Corning). After 2 days, the medium was replaced with fresh medium containing 1 μm dexamethasone for 30 min to synchronize the cells. This was washed three times with PBS and replaced with fresh medium. Two wells were immediately plugged with size 4 silicone rubber stoppers (Saint‐Gobain, Courbevoie, France), the bottoms of which were cut off such that the bottoms of the stoppers were just below the top of the well to leave room for air above the medium. Two 18‐gauge needles (BD PrecisionGlide, Franklin Lakes, NJ) were inserted vertically through each stopper on opposite ends of the well and were pushed down until the tips touched the bottom of the well. Medium‐filled syringes were loaded into a syringe pump outside the incubator and were attached to the inlet needles of each well by 1 m of silicone tubing, which allowed heat and CO_2_ to enter the medium stream before reaching the cells. The outlet needles of the wells were each connected to a fraction collector by silicone tubing. To initially fill all of the tubing, medium was pumped from the syringe pump at 1 mL·min^−1^ until medium started dispensing out of the fraction collector, at which point the flow rate was switched to 1 mL·h^−1^, and the syringe pumps began collecting 1 fraction per hour After 24 h, fractions were immediately frozen at −20 °C until assayed for GLUC as described above.

### RT‐qPCR

Jurkat cells were synchronized as described above then seeded in a 24‐well plate at 1 × 10^6^ cells/well, and one well was harvested and washed 30 min and then every 2 h post synchronization for 24 h. Cells were snap frozen and stored in N2(l) until all cells were collected. Samples for each replicate set were processed simultaneously. RNA was isolated from each sample using the PureLink RNA Mini Kit (Thermo cat # 12183025) and A260/280 ratios were determined using a NanoDrop ND‐1000 (Thermo). Each sample was used to generate cDNA using the iScript cDNA Synthesis Kit (Bio‐Rad cat # 170‐8891), and the PowerUp SYBR Green Master Mix (Applied Biosystems cat # A25742, Foster City, CA) was used to perform qPCR according to the manufacturer’s instructions using a viia 7 Real‐Time PCR System (Applied Biosystems). Primer sequences can be found in Table S1. C*_t_* values were automatically generated by the viia 7 software. The ΔCt values of each gene at each time point corresponds to the average C*_t_* value subtracted by the average C*_t_* value of GAPDH at the same time point, then transformed by $2^{‐\Delta C_{t}}$. For heat map representation and statistical analysis, each gene’s data set between 2 and 20 h postsynchronization was normalized to the lowest value of the set. To determine whether the lowest and highest values (presumably peaks and troughs) were statistically significant, *q*‐values were calculated using prism 8 (GraphPad).

### Animal procedures

In all experiments, female NSG (NOD.Cg‐Prkdc^scid^ Il2rg^tm1Wjl^/SzJ, Jackson Laboratory stock # 005557) mice 3–6 months old were used, and they were housed in a 12 h on/12 h off facility fed *ad libitum*. Jurkat cells were washed 4–5 times with PBS and resuspended in 500 μL PBS before their intraperitoneal injection at 5 × 10^5^ stably selected cells per animal. For all experiments, < 10% of the animal’s total blood volume was drawn by tail snip within a 24‐h period around 40 days post injection. Immediately upon drawing blood, heparin sodium (McKesson Corporation cat # 916396, Irving, TX) was added at a final concentration of 0.002 U·mL^−1^ and spun at 2000 ***g*** for 10 min at 4 °C. The plasma supernatant was stored −80 °C. Using methods described above, 5 μL of plasma was assayed for GLUC or CLUC activity. Animals were euthanized when they showed clear signs of engraftment related disease as compared to control animals. All animal work was performed in accordance with the ethical standards of the Institutional Animal Care and Use Committee.

### Data analysis and statistics

All line fitting was performed using prism 8 (GraphPad). Linear detrending and period determination of circadian data was performed using BioDare2 [50]. For Fig. 3F, an undamped 24‐h Sine wave was fitted to the data (Prism, least squares method, degrees of freedom: 14, R squared: 0.7777, sum of squares: 0.4124). To determine whether the lowest and highest values (presumably peaks and troughs) were statistically significant, *q*‐values were calculated using prism 8 (GraphPad).

## Conflict of interest

A patent on engineered cell technology and uses for diagnostics and therapeutics has been filed. The authors declare no conflict of interest.

## Author contributions

BP, AGT, AB involved in the conceptualization; AGT, AB and SS involved in the execution of experiments; AGT, AB, and BP performed the formal analysis; AGT, AB, and BP performed the manuscript preparation; BP involved in the funding acquisition.

## Supporting information


**Fig. S1**. Sensitivity of GLUC luciferase detection secreted from cells and purified GLUC. (A) EF1α‐GLUC cells seeded at various densities were incubated for 1 or 3 h, and conditioned media was assayed for GLUC secretion. Inset contains lower cell densities. Linear regression fit, R squared = 0.9934. (B) Standard curve of GLUC. Inset contains lower GLUC amounts. Linear regression fit, R squared = 0.9938.

**Fig. S2**. Dynamic expression of GLUC from Circa2 in U2OS cells. U2OS cell secretions were collected from synchronized cells expressing a Circa2‐Gluc construct and assayed GLUC.

**Fig. S3**. Jurkat cell engineering with recombinant DNA and effects on growth rate. (A) Untransduced (upper top panels) versus transduced (lower panels) leukemic T cells with a GFP selective marker. Scale bars = 400 µm (B) Growth rates of untransduced versus EF1α‐GLUC cells. Starting densities of each cell type were different as noted on the Y‐axis at time 0. Linear regression fit, R squared = 0.9870 and 0.9508 respectively.

**Fig. S4**. Endogenous circadian gene expression in Jurkat leukemic T cells. Synchronized Jurkat cells were collected approximately every 2 h and RNA was isolated. RT‐qPCR using primers specific for (A) Per1, (B) Per2, (C) Cry1, (D) Cry2, (E) Bmal1 and (F) Clock was performed and the ΔCt was calculate using GAPDH a reference gene (SD; *N* = 3). (G) The lowest ΔCt values were normalized (to 1) for each gene. (H) Differences between lowest and highest normalized ΔCt values for each gene were statistically significant if *q*‐value was </= 0.001 (‐log>/= 2).

**Fig. S5**. Underlying data for constitutive and circadian clock reporter dynamics of leukemic T cells infiltrating immuno‐compromised mice. Blood was collected approximately every 4 h from mice injected with either EF1α‐GLUC (A) or Circa2‐GLUC (B).EF1α‐CLUC and Circa2‐GLUC Jurkat cells were co‐injected into a single mouse, then blood was collected every 4 h and assayed for CLUC (C) and GLUC (D). Lights on at ZT0 and lights off at ZT12. Raw RLU counts ar shown (5 μL blood). Each line represents a single mouse.

**Fig. S6**. Continuous flow system media collection system. Shown is the air permeable vessel containing cells in the blue rack, connected to tubing leading from media syringes on pump (left) and tubing which leads out of the back of the incubator to a fraction collector (right).

**Table S1**. DNA Sequences. All sequences shown in 5’ to 3’. E‐box sequences are underlined and minimal promoters are in bold. Forward and reverse RT‐qPCR primers are shown paired.

## Data Availability

Data will made availability from the corresponding author upon reasonable request.
