# Supplementary figures and images for: Tracking leukemic T‐cell transcriptional dynamics in vivo with a blood‐based reporter assay
Source: FEBS Open Bio. 2020 Aug 12;10(9):1868–79. doi: 10.1002/2211-5463.12940 (PMC7459418; doi:10.1002/2211-5463.12940)

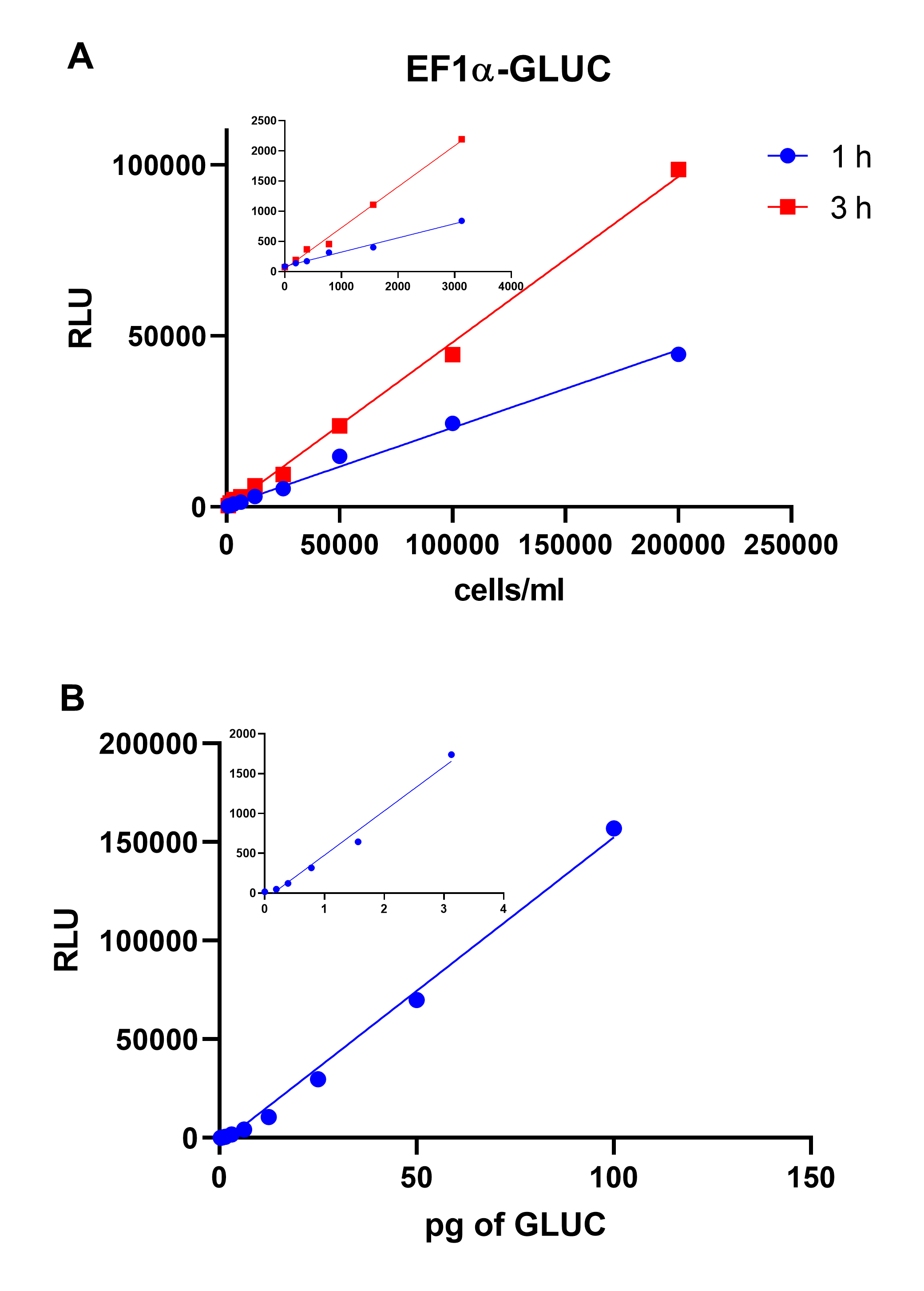

Supplement: Supplementary file 1 — Fig. S1. Sensitivity of GLUC luciferase detection secreted from cells and purified GLUC. (A) EF1α‐GLUC cells seeded at various densities were incubated for 1 or 3 h, and conditioned media was assayed for GLUC secretion. Inset contains lower cell densities. Linear regression fit, R squared = 0.9934. (B) Standard curve of GLUC. Inset contains lower GLUC amounts. Linear regression fit, R squared = 0.9938. [file FEB4-10-1868-s001.tif]

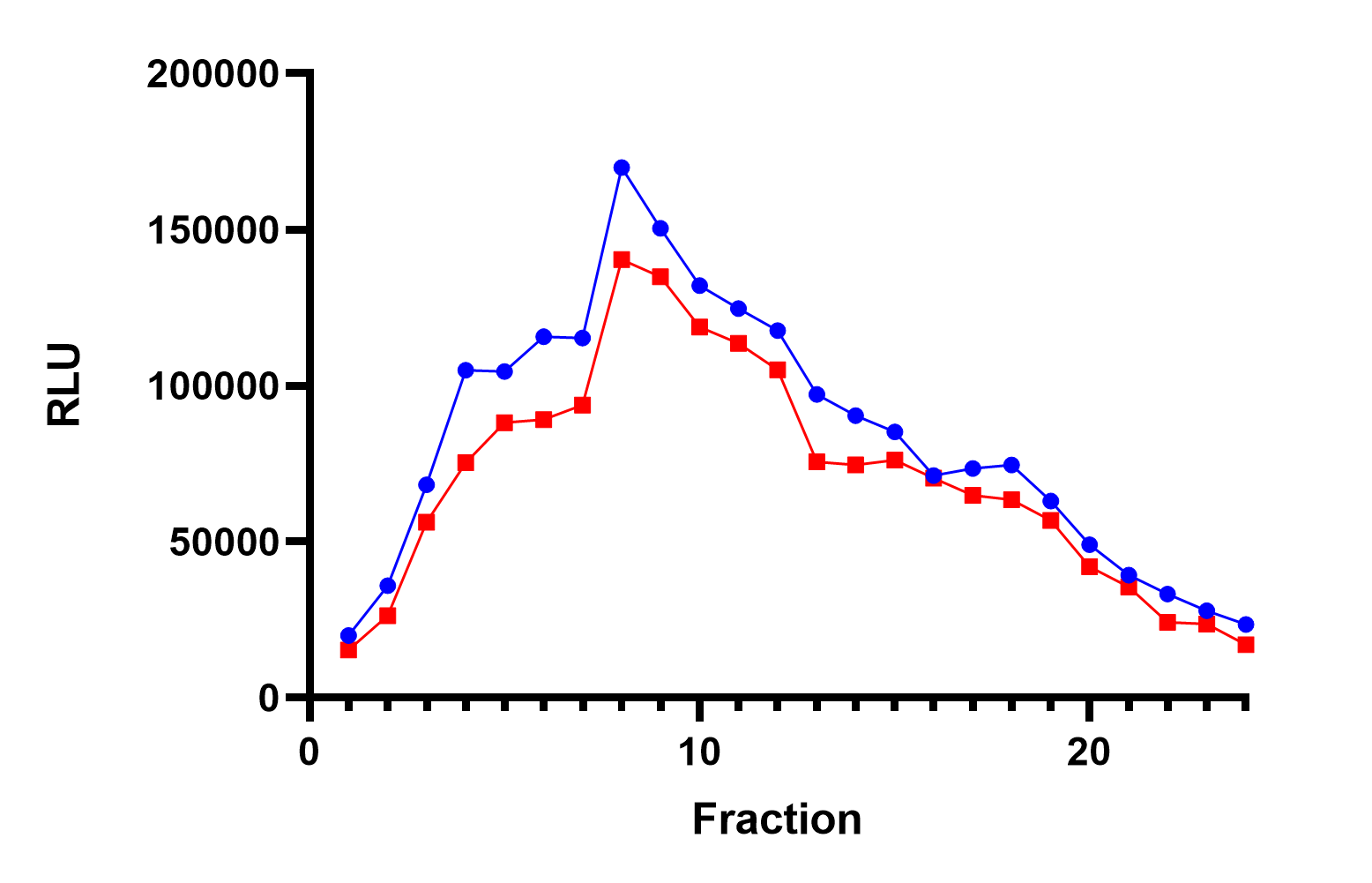

Supplement: Supplementary file 2 — Fig. S2. Dynamic expression of GLUC from Circa2 in U2OS cells. U2OS cell secretions were collected from synchronized cells expressing a Circa2‐Gluc construct and assayed GLUC. [file FEB4-10-1868-s002.tif]

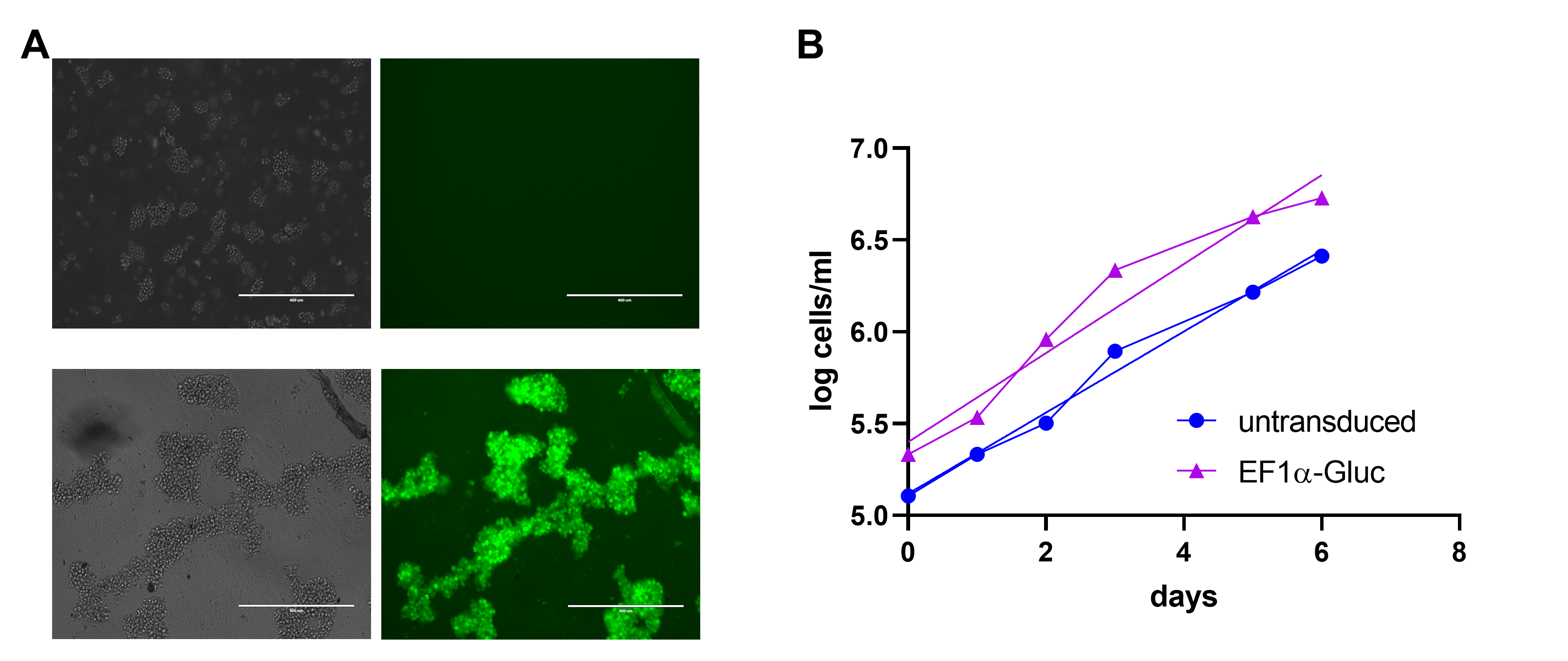

Supplement: Supplementary file 3 — Fig. S3. Jurkat cell engineering with recombinant DNA and effects on growth rate. (A) Untransduced (upper top panels) versus transduced (lower panels) leukemic T cells with a GFP selective marker. Scale bars = 400 µm (B) Growth rates of untransduced versus EF1α‐GLUC cells. Starting densities of each cell type were different as noted on the Y‐axis at time 0. Linear regression fit, R squared = 0.9870 and 0.9508 respectively. [file FEB4-10-1868-s003.tif]

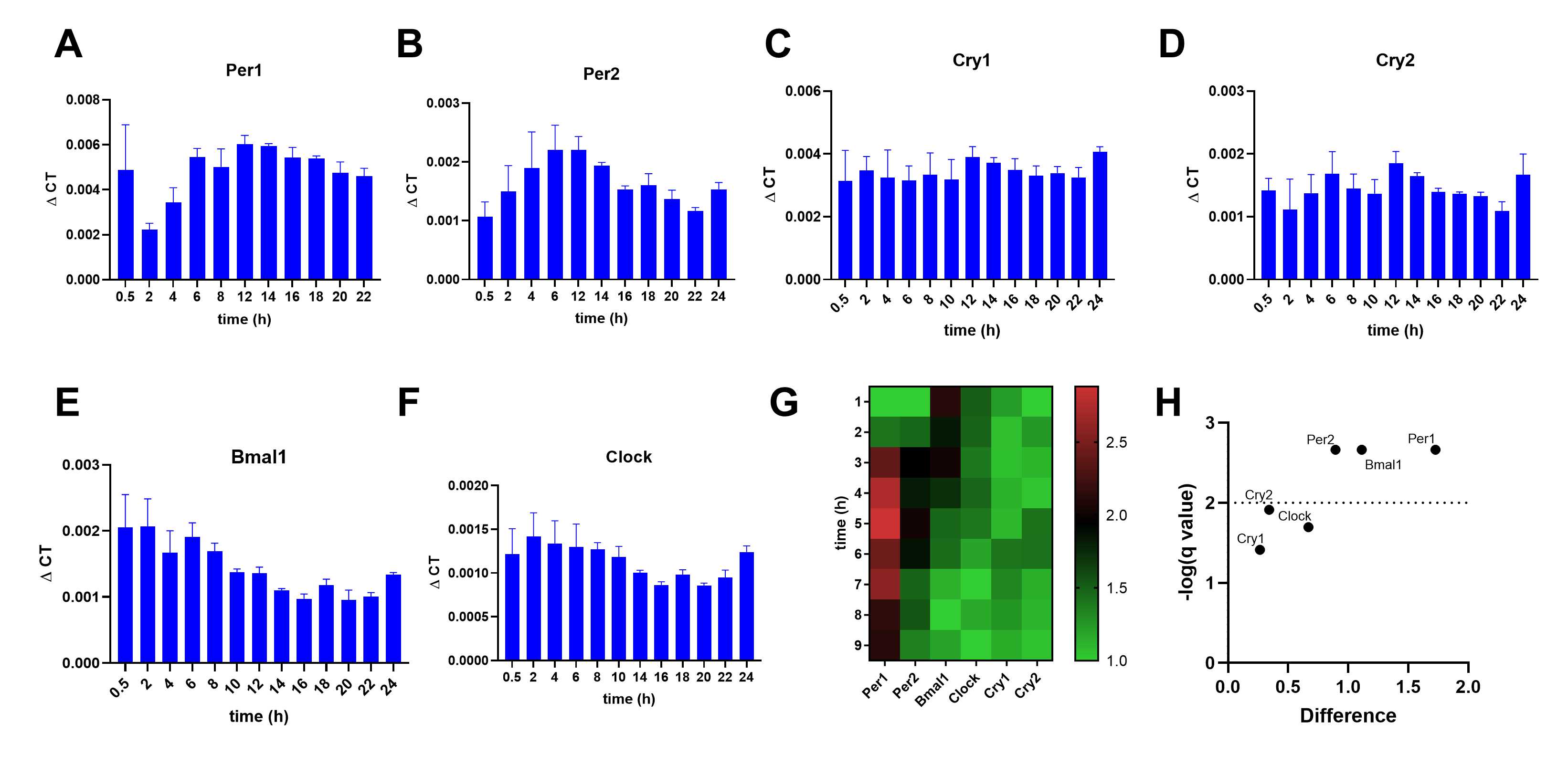

Supplement: Supplementary file 4 — Fig. S4. Endogenous circadian gene expression in Jurkat leukemic T cells. Synchronized Jurkat cells were collected approximately every 2 h and RNA was isolated. RT‐qPCR using primers specific for (A) Per1, (B) Per2, (C) Cry1, (D) Cry2, (E) Bmal1 and (F) Clock was performed and the ΔCt was calculate using GAPDH a reference gene (SD; N = 3). (G) The lowest ΔCt values were normalized (to 1) for each gene. (H) Differences between lowest and highest normalized ΔCt values for each gene were statistically significant if q‐value was </= 0.001 (‐log>/= 2). [file FEB4-10-1868-s004.tif]

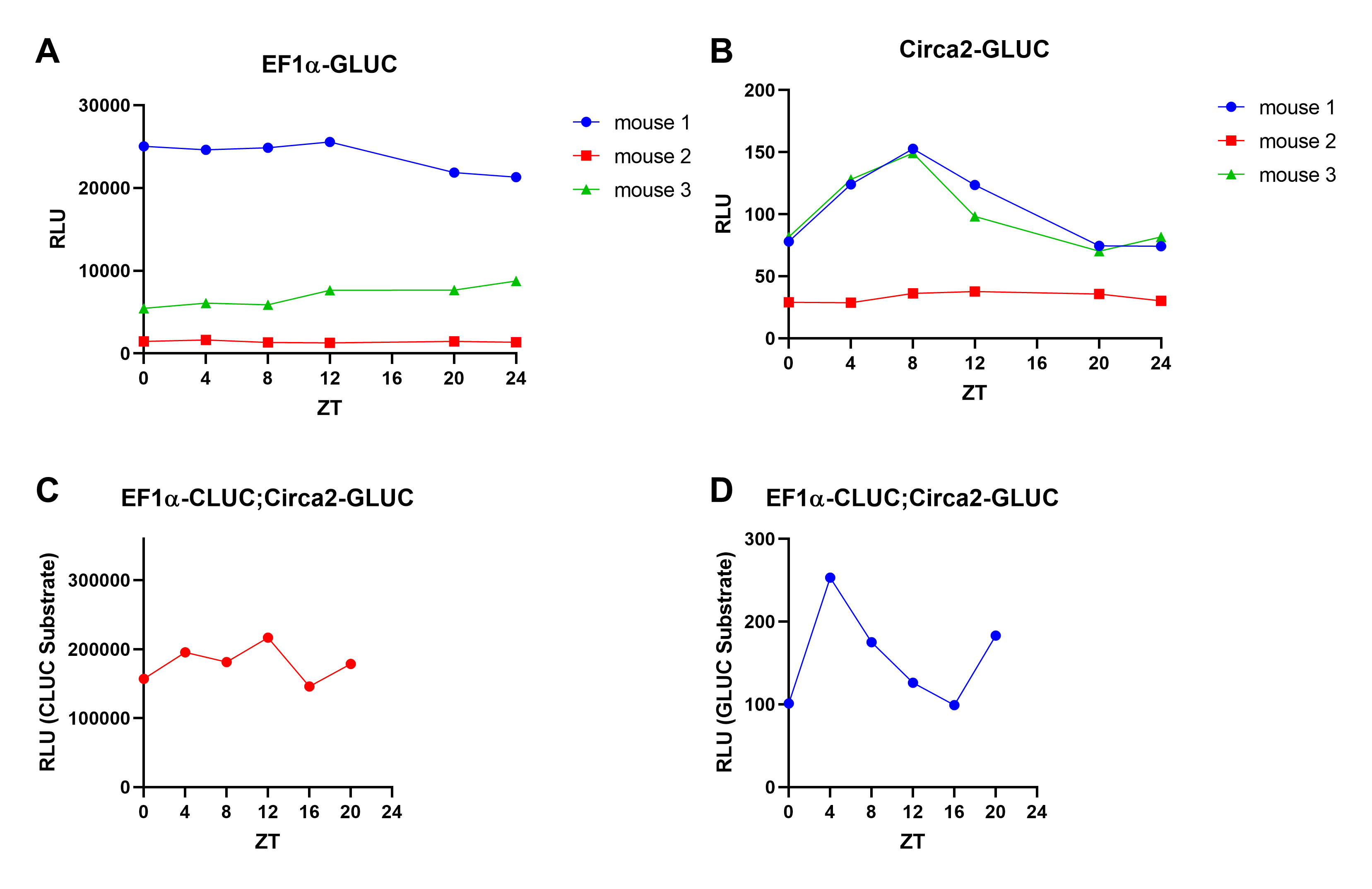

Supplement: Supplementary file 5 — Fig. S5. Underlying data for constitutive and circadian clock reporter dynamics of leukemic T cells infiltrating immuno‐compromised mice. Blood was collected approximately every 4 h from mice injected with either EF1α‐GLUC (A) or Circa2‐GLUC (B).EF1α‐CLUC and Circa2‐GLUC Jurkat cells were co‐injected into a single mouse, then blood was collected every 4 h and assayed for CLUC (C) and GLUC (D). Lights on at ZT0 and lights off at ZT12. Raw RLU counts ar shown (5 μL blood). Each line represents a single mouse. [file FEB4-10-1868-s005.tif]

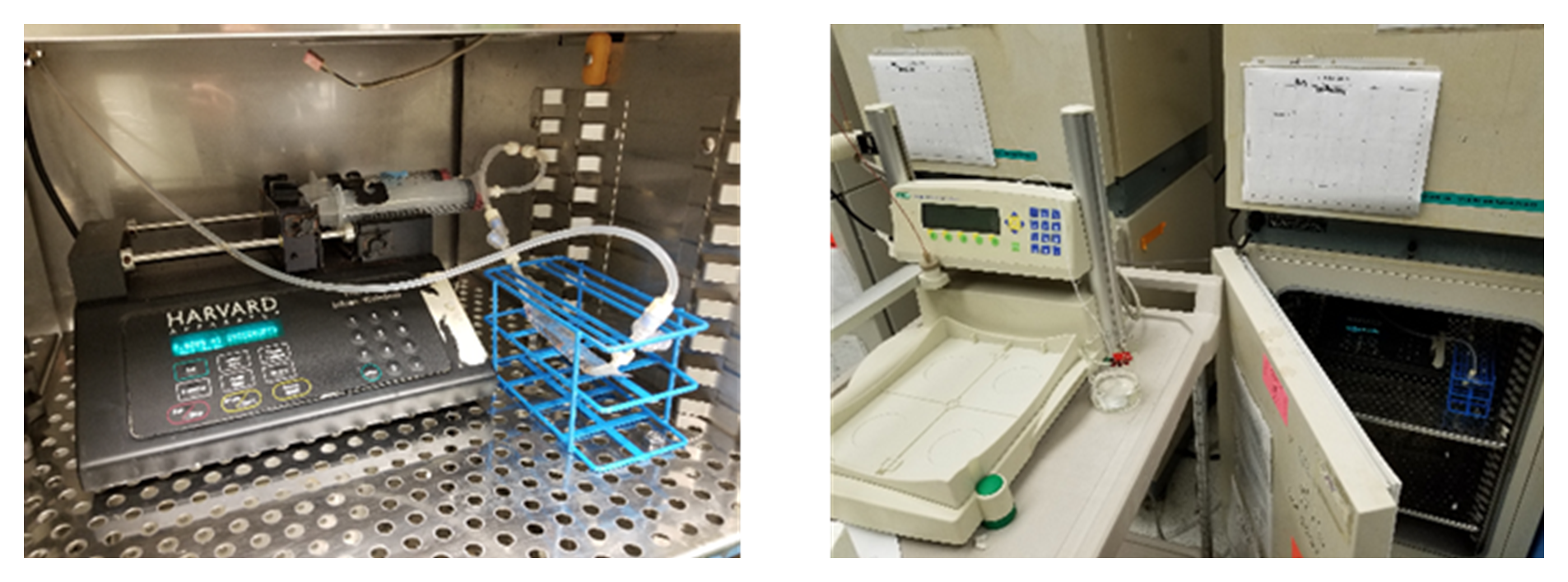

Supplement: Supplementary file 6 — Fig. S6. Continuous flow system media collection system. Shown is the air permeable vessel containing cells in the blue rack, connected to tubing leading from media syringes on pump (left) and tubing which leads out of the back of the incubator to a fraction collector (right). [file FEB4-10-1868-s006.tif]
