# Supplementary material for: Tracking leukemic T‐cell transcriptional dynamics in vivo with a blood‐based reporter assay
Source: FEBS Open Bio. 2020 Aug 12;10(9):1868–79. doi: 10.1002/2211-5463.12940 (PMC7459418; doi:10.1002/2211-5463.12940)
Supplement: Supplementary file 7 — Table S1. DNA Sequences. All sequences shown in 5’ to 3’. E‐box sequences are underlined and minimal promoters are in bold. Forward and reverse RT‐qPCR primers are shown paired. [file FEB4-10-1868-s007.docx]

Table S1: DNA Sequences

| Circa1 | AGTAGTGTTAACCCCGGGCTCGAGCAGTATTTAGCCACGTGACAGTGTAAGCACACGTGGGCCCTCAAGTCCACGTGCAGGGAGCCTGAGCACCACTGAGCGGTCCTGAGCCA**AGAGGGTATATAATGGAAGCTCGACTTCCAG** |
| --- | --- |
| Circa2 | AGTAGTGTTAACCCCGGGCTCGAGCAGTATTTAGCCACGTGACAGTGTAAGCACACGTGGGCCCTCAAGTCCACGTGCAGGGAGCCACGTGACCACACGTGGGTCCACGTGCA**AGAGGGTATATAATGGAAGCTCGACTTCCAG** |
| Per1 | TCAACTGCCTGGACAGCATCCT  TCAGAGGCTGAGGAGGTGGTAT |
| Per2 | AGCTGCTTGGACAGCGTCATCA  CCTTCCGCTTATCACTGGACCT |
| Cry1 | GCAGTTGCTTGCTTCCTGACAC  GACAGCCACATCCAACTTCCAG |
| Cry2 | AGGAGAACCACGACGAGACCTA  CCGTTCCAAGTGCTTATCCAGG |
| Clock | CAGGCAGCATTTACCAGCTCATG  GTAGCTTGAGACATCACTGGCTG |
| Bmal1 | GCTCAGGAGAACCCAGGTTATC  GCATCTGCTTCCAAGAGGCTCA |
| Gapdh | GTCTCCTCTGACTTCAACAGCG  ACCACCCTGTTGCTGTAGCCAA |
| β-Actin | CACCATTGGCAATGAGCGGTTC  AGGTCTTTGCGGATGTCCACGT |
